# Estimating utility weights and quality-adjusted life year loss for colorectal cancer-related health states in Korea

**DOI:** 10.1038/s41598-017-06004-6

**Published:** 2017-07-17

**Authors:** Jin Yong Lee, Minsu Ock, Min-Woo Jo, Woo-Seung Son, Hyeon-Jeong Lee, Seon-Ha Kim, Hyun Joo Kim, Jong Lyul Lee

**Affiliations:** 1 Public Health Medical Service, Boramae Medical Center, Seoul National University College of Medicine, Seoul, Republic of Korea; 2 Institute of Health Policy and Management, Medical Research Center, Seoul National University College of Medicine, Seoul, Republic of Korea; 3 Department of Preventive Medicine, Ulsan University Hospital, University of Ulsan College of Medicine, Ulsan, Republic of Korea; 4 Department of Preventive Medicine, University of Ulsan College of Medicine, Seoul, Republic of Korea; 5 Department of Nursing, College of Nursing, Dankook University, Cheonan, Republic of Korea; 6 Department of Nursing Science, Shinsung University, Dangjin, Republic of Korea; 7 Departments of Colon and Rectal Surgery, Asan Medical Center, University of Ulsan College of Medicine, Seoul, Republic of Korea

## Abstract

We aimed to assess utility weight of health states associated with colorectal cancer (CRC) that reflect the societal preference of the Korean population and to estimate the quality-adjusted life year (QALY) loss with CRC. We recruited 607 individuals from the Korean population; they were surveyed via face-to-face computer-assisted interviews. The participants evaluated each CRC-associated health state using standard gamble. Utility weight for each health state was calculated as the possibility of full health restoration. Moreover, we estimated total QALY loss due to CRC in Korean individuals aged ≥30 years in 2013. To calculate QALY due to morbidity, we yielded utility weights and used epidemiologic data of CRC on severity from the National Cancer Control Institute. QALY loss due to mortality was calculated using mortality of CRC and life expectancy data from the Korean Statistical Information Service. The highest and lowest utility weights were assigned to “adenomatous polyps” and “metastatic colon cancer”, respectively. Total QALY loss due to CRC in Korea was 173,662; these patients were more likely to be men or be included in the 70–74-year age group. These utility weights may be useful for conducting cost-utility studies of cancer screening for CRC and for measuring disease burden with QALY.

## Introduction

Colorectal cancer (CRC) is the fourth most-common cause of cancer-related death, and has the third highest incidence rate among malignant neoplasms in South Korea (hereinafter referred to as Korea). In 2012, a total of 8,135 patients have died due to CRC and 28,988 patients have been newly diagnosed with CRC, which can lead to a considerable disease burden in individuals, families, and societies^1^.

Several methods are available to calculate the burden of disease (BoD). Traditionally, mortality and life expectancy have been used to calculate BoD. However, at present, summary measures such as quality-adjusted life-year (QALY) or disability-adjusted life-year (DALY), which reflect both the quantity and quality of life, are commonly used worldwide^2^. QALY is a single index generated from combining disease mortality and morbidity, and is used as the official measurement scale of The National Institute for Health and Clinical Excellence (NICE) in the United Kingdom^3–5^.

QALY is widely used as a measurement tool for health effectiveness and can assist decision makers in setting priorities among competing healthcare programs in situations with limited resources^6–8^. In particular, QALY can provide a common currency value for the benefits or gains from healthcare interventions. QALYs can be calculated simply by multiplying the duration spent in a health state (HS) by the health-related quality of life (HRQoL) weight (i.e. utility weight for HS) associated with that HS^9^. Hence, in order to calculate QALYs, it is essential to first obtain the utility weights (or quality weights) of various HSs^3^.

With regard to the estimation of HS utility weight, there are two important issues. First, the individuals whose preferences should be reflected need to be identified; for instance, the parameter can be estimated from the general population, patients, healthcare professionals, or government. Second, the type of valuation technique to be used needs to be determined. With regard to the first issue, many researchers have recently suggested that the societal preference is more appropriate^10, 11^. With regard to the valuation methods, the direct ones, including the visual analogue scale (VAS), time trade-off (TTO), and standard gamble (SG), are widely used for eliciting preferences^12^. In oncology, some studies have measured the utility weights of described elicited HSs from the general population by using SG or TTO^13, 14^. SG is the gold standard for eliciting expected utilities^15^ and could be feasible, reliable, and valid method to value HSs in Korea^16^. Finally, due to the cultural differences among countries, the health utility scores should be evaluated in the context of their own societal values^17^.

We believe that the BoD due to CRC may be considerable in Korea. However, little is known about the utility weights of CRC in Korea. In the present study, we aimed to determine the utility weights of HSs associated with CRC that reflect the societal preference of the Korean population using SG and estimate the QALY loss due to CRC.

## Results

Table 1 presents general information regarding the study participants eliciting utility weights for CRC-related HSs using SG, including gender, age group, education level, occupation, monthly incomes, and related clinical features.

**Table 1:** General characteristics of the study participants.

Variable		N	%
Gender	Man	281	49.9
	Woman	282	50.1
Age group (years)	19–29	104	18.5
	30–39	110	19.5
	40–49	118	21.0
	50–59	111	19.7
	≥60	120	21.3
Education level	Elementary school or below	20	3.6
	Middle school	44	7.8
	High school	363	64.5
	College or above	136	24.2
Occupation	Non-manual	145	25.8
	Manual	271	48.2
	Other	146	26.0
Monthly income	<2.5 million won	122	21.7
	2.5–5.0 million won	378	67.1
	≥5.0 million won	63	11.2
Ambulatory care visit in the past 2 weeks	Yes	63	11.2
	No	500	88.8
Hospitalization in past 12 months	Yes	18	3.2
	No	545	96.8
Morbidity	Yes	53	9.4
	No	510	90.6

### Utilities of health states associated with CRC according to SG

The highest utility weight was derived from HS 1 (0.81), followed by HS 2 (0.69) and HS 3 (0.66), whereas the lowest utility weight was derived from HS 7 (0.45). For each HS, except for HS 7, the mean utility weights calculated via SG did not significantly differ according to gender, age group, education level, occupation, monthly incomes, and related clinical features (Table 2).

**Table 2:** Utility weights by health states related to colorectal cancer.

Variable		HS 1	HS 2	HS 3	HS 4	HS 5	HS 6	HS 7
Total	N	415	402	396	407	422	392	381
	Mean ± SD	0.76 ± 0.31	0.67 ± 0.32	0.62 ± 0.33	0.57 ± 0.32	0.59 ± 0.31	0.57 ± 0.31	0.47 ± 0.32
	Median	0.90	0.80	0.75	0.65	0.65	0.65	0.45
	1st quartile	0.60	0.35	0.30	0.30	0.30	0.30	0.20
	2nd quartile	0.90	0.80	0.75	0.65	0.65	0.65	0.45
	3rd quartile	1.00	0.95	0.90	0.85	0.85	0.84	0.75
Gender	Man	0.82	0.72	0.70	0.62	0.62	0.61	0.47
	Woman	0.79	0.67	0.61	0.57	0.59	0.57	0.43
Age group (years)	19–29	0.78	0.73	0.66	0.63	0.63	0.63	0.49
	30–39	0.81	0.64	0.64	0.56	0.60	0.58	0.46
	40–49	0.84	0.68	0.68	0.59	0.62	0.60	0.42
	50–59	0.80	0.71	0.67	0.58	0.60	0.56	0.43
	≥60	0.81	0.71	0.63	0.63	0.60	0.58	0.46
Education level	High school or below	0.81	0.69	0.64	0.60	0.59	0.57	0.43
	College or above	0.81	0.69	0.68	0.59	0.64	0.62	0.48
Occupation	Non-manual	0.82	0.68	0.69	0.62	0.63	0.62	0.53**
	Manual	0.78	0.69	0.64	0.56	0.59	0.55	0.38**
	Other	0.84	0.71	0.65	0.64	0.63	0.62	0.50**
Monthly income	<2.5 million won	0.81	0.69	0.64	0.62	0.58	0.57	0.34*
	2.5–5.0 million won	0.80	0.68	0.64	0.57	0.60	0.58	0.48*
	≥5.0 million won	0.88	0.76	0.78	0.72	0.71	0.72	0.48*
Ambulatory care visit in the past 2 weeks	Yes	0.79	0.68	0.68	0.58	0.60	0.58	0.35
	No	0.81	0.69	0.65	0.60	0.61	0.59	0.46
Hospitalization in the past 12 months	Yes	0.84	0.74	0.75	0.68	0.60	0.63	0.30
	No	0.81	0.69	0.65	0.60	0.61	0.59	0.46
Morbidity	Yes	0.80	0.69	0.70	0.64	0.59	0.56	0.42
	No	0.81	0.69	0.65	0.59	0.61	0.59	0.45

*P-value < 0.05, **P-value < 0.01.

HS: health state; SD: standard deviation.

HS1: Adenomatous polyps; HS2: Colon cancer requiring colon resection; HS3: Rectal cancer requiring resection of the rectum; HS4: Colon cancer requiring colon resection and systemic chemotherapy; HS5: Rectal cancer requiring resection of the rectum and chemoradiation therapy; HS6: Rectal cancer requiring resection of the rectum, stoma formation, and chemoradiation therapy; HS7: Metastatic colon cancer.

### QALY losses due to health states related to CRC

The total QALY loss due to CRC in Korea in 2013 was 173,662. Estimates of total QALY loss due to CRC by gender and age group are shown in Table 3. The total QALY loss due to CRC was larger in men (98,046) than in women (75,616). Among the age groups, the total QALY loss due to CRC was greatest in the 70–74-year age group (25,225), followed by the 60–64-year age group (22,002) and the 65–69-year age group (21,339). In terms of the Surveillance, Epidemiology, and End Results (SEER) stage, the QALY loss due to CRC morbidity was greatest in the regional stage (19,088), followed by the localized stage (15,576) and distance stage (5,482). The results from sensitivity analyses were shown in the Supplementary Tables 1 and 2.

**Table 3:** QALY loss due to colorectal cancer by gender and age group in Korea in 2013.

Gender	Age group	QALY loss due to morbidity (by SEER stage)					QALY loss due to mortality
		Localized (95% CI)	Regional (95% CI)	Distance (95% CI)	Unknown (95% CI)	Total (95% CI)	
Man	30–34	124 (111–136)	79 (73–85)	35 (33–37)	24 (22–26)	262 (239–284)	1,266
	35–39	258 (232–284)	173 (160–187)	65 (61–69)	58 (54–62)	554 (507–602)	1,389
	40–44	415 (374–457)	374 (346–403)	124 (117–132)	87 (81–93)	1,000 (918–1,085)	3,402
	45–49	664 (599–731)	672 (621–724)	217 (204–231)	142 (132–152)	1,695 (1,556–1,838)	5,084
	50–54	1,277 (1,151–1,407)	1,297 (1,200–1,398)	389 (365–413)	231 (216–247)	3,194 (2,932–3,465)	8,486
	55–59	1,429 (1,288–1,574)	1,596 (1,477–1,719)	475 (446–505)	258 (241–276)	3,758 (3,452–4,074)	8,969
	60–64	1,630 (1,469–1,795)	1,821 (1,684–1,961)	487 (457–517)	286 (267–305)	4,224 (3,877–4,578)	9,820
	65–69	1,558 (1,405–1,717)	1,806 (1,671–1,945)	486 (456–516)	280 (261–299)	4,130 (3,793–4,477)	8,849
	70–74	1,348 (1,215–1,485)	1,835 (1,697–1,976)	465 (437–494)	264 (247–282)	3,912 (3,596–4,237)	11,379
	75–79	736 (663–811)	1,119 (1,035–1,205)	287 (269–305)	212 (198–227)	2,354 (2,165–2,548)	7,013
	80+	391 (352–430)	622 (576–670)	199 (187–211)	271 (253–289)	1,483 (1,368–1,600)	5,827
	Total	9,828 (8,860–10,826)	11,392 (10,540–12,273)	3,230 (3,030–3,430)	2,113 (1,972–2,258)	26,563 (24,402–28,787)	71,483
Woman	30–34	70 (63–77)	80 (74–86)	35 (33–37)	15 (14–16)	200 (184–216)	958
	35–39	161 (145–177)	148 (137–159)	52 (49–55)	31 (29–33)	392 (360–424)	1,692
	40–44	263 (237–289)	304 (282–328)	124 (117–132)	48 (44–51)	739 (680–800)	2,306
	45–49	408 (368–450)	490 (453–528)	193 (181–205)	92 (86–99)	1,183 (1,088–1,282)	4,337
	50–54	733 (660–807)	878 (812–946)	251 (235–266)	124 (116–133)	1,986 (1,823–2,152)	6,898
	55–59	718 (648–791)	865 (800–931)	238 (223–252)	131 (122–140)	1,952 (1,793–2,114)	5,682
	60–64	771 (695–849)	889 (822–957)	246 (231–262)	134 (125–143)	2,040 (1,873–2,211)	5,918
	65–69	801 (722–882)	1,096 (1,014–1,181)	258 (242–274)	137 (128–146)	2,292 (2,106–2,483)	6,068
	70–74	833 (751–917)	1,190 (1,101–1,282)	312 (293–331)	178 (167–191)	2,513 (2,312–2,721)	7,421
	75–79	585 (528–645)	981 (907–1,057)	268 (251–284)	210 (196–224)	2,044 (1,882–2,210)	7,872
	80+	406 (366–448)	776 (718–835)	276 (259–293)	451 (421–482)	1,909 (1,764–2,058)	9,217
	Total	5,748 (5,182–6,332)	7,696 (7,120–8,291)	2,252 (2,113–2,391)	1,551 (1,448–1,658)	17,247 (15,863–18,672)	58,369

Quality-adjusted life year (QALY); Confidence interval (CI).

## Discussion

In the present study, we aimed to determine the utility weights of HSs associated with CRC that reflect the societal preference of the Korean population, and to estimate the QALY loss due to CRC. The highest utility weight was derived from HS 1 (0.81), whereas the lowest weight was derived from HS 7 (0.45). In 2013, the total QALY loss due to CRC in Korea was 173,662; in particular, men (98.046) had a larger QALY loss than women (75,616), and patients aged 70–74 years (25,225) had the highest QALY loss, as compared to the others. In terms of the SEER stage, the QALY loss due to CRC morbidity was greatest in the regional stage (19,088), followed by the localized stage (15,576) and the distance stage (3,664).

We used QALY as an indicator of the value of health outcomes in our current study. A health utility weight is required to yield the QALY value. With regard to health utility, there are two main issues. The first issue includes the individuals whose preference should be considered. Although the use of patients’ or providers’ perspective has its own significance, the societal perspective is emphasized in economic evaluation guidelines^18^. In Korea, the use of the societal perspective is recommended in the guidelines for economic evaluation of pharmaceuticals, as compared to the patients’ or providers’ perspective^11^. Furthermore, Gabriel *et al*. reported that cost-utility studies based on the patients’ perspective may undervalue prevention interventions, as patients tend to value their HS more favourably than those who have not experienced the illness^19^. In the study of Best *et al*., the preference values for HSs related to Stage III colon cancer were higher in patients than in community members^20^. Therefore, the general consensus is that the preferences of the general population should carry more weight than those of patients or providers^10^. Thus, we used the preferences from the general population in Korea to estimate the utility weights for CRC-related HSs in this study.

The other issue is the type of utility evaluation technique to be used. The valuation methods can be categorized as direct or indirect^12^. For the indirect valuation method, preference-based instruments, such as European quality of life 5D (EQ-5D), short form 6D (SF-6D), and health utilities index (HUI), are usually used. For the direct valuation method, SG, TTO, and VAS are commonly used. Although there is no generally accepted theoretical basis for selecting direct or indirect valuation methods, we used the SG method in our study because it measures participants’ preferences under conditions of uncertainty, and is directly based on the von Neumann-Morgenstern utility theory, which is regarded as the gold standard for modelling rational behaviour in the context of uncertainty^21^.

The utility weights estimated from this study using SG did not significantly differ according to gender, age group, and related clinical features. Furthermore, except for HS 7, there was no statistically significant difference between or among utility weights according to education level, type of occupation, and monthly income. We assumed that the HS descriptions dominantly affected the participants’ preference, regardless of socio-demographic factors, such as gender and age. Furthermore, in general, it seemed that the utility weights calculated in the present study were lower than those calculated in other studies^22^. According to a systematic review study, the utility weights of HSs associated with CRC differ markedly between studies and across methods. Five studies reported the utility weights of HSs according to the severity of CRC, similar to that in the present study. The ranges of the utility weights by stage were 0.74–0.84 (stage I), 0.59–0.86 (stage II), 0.59–0.82 (stage III), and 0.25–0.84 (stage IV)^22^. Among the five studies, only one study used a direct valuation method and obtained utility weights as follows: 0.74 (stage I rectal cancer or stage I/II colon cancer), 0.67 (stage III colon cancer), 0.59 (stage II/III rectal cancer without ostomy), 0.50 (stage II/III rectal cancer with ostomy), and 0.25 (stage IV rectal cancer or colon cancer)^23^. In general, the results from Ness *et al*. were similar to our present results. The other four studies used indirect valuation methods, and their utility weights by CRC severity were relatively higher than those noted in the present study. These differences might be driven by methodological differences. In case of using direct method such as SG, their utility weights might be underestimated because there was no adaptation process on their HSs. Generally, cancer patients have gone through process in order to accept their illness but in eliciting a stated preference people could not undergo but just image these states. So they could valuate states worse than those real states. On the other hand, in indirect approach, utility weights might be overestimated because some patients with better conditions more easily could response to surveys. Utility weights from indirect approaches could be overestimated.

In addition, there were some inconsistencies in terms of CRC severity in other studies, whereas the utility weights estimated in our present study were consistent. For example, Wilson *et al*. reported that the EQ-5D index for Dukes stage A + B was 0.786, but that of Dukes stage C + D was 0.806^24^. Furthermore, Wong *et al*. determined that the mean SF-6D health preference scores were 0.831 in stage I and 0.858 in stage II, respectively^25^. We believe that there are two main factors explaining these inconsistencies. First, the inconsistencies may be due to the small sample size. We believe that the consideration of the preferences from a relatively large sample (n = 607) in our current study may have played a role in the consistent utility weights obtained according to CRC. Hence, a utility study should have a sufficient sample size in order to obtain consistent values according to the severity level. Second, the differences in the HRQoL among patients with mild HSs may not be remarkable. Therefore, the perspectives of patients need to be compared to those from the general public in utility weight studies for mild HSs.

There were also some inconsistent responses in individual levels, however this inconsistent rate could be acceptable. In this study, among 607 participants, 419 participants (69.0%) conducted SG tasks without any inconsistency and 10.0% had more than four inconsistencies in 11 pairs (Supplementary Table 3). The Korean EQ-5D-5L valuation study using composite TTO reported 66.4% of consistent respondents^26^ although there were some differences in health state description and valuation method between two studies. Considering that TTO can be relatively easier than SG, it might show that SG tasks in this study were appropriately conducted in the perspective of inconsistency. In addition, each standard deviation of HS’s utility weight was reduced by exclusion of inconsistent responses, respectively. In this study, we allowed arbitrarily three inconsistent responses.

The utility weight is not simply an auxiliary tool for measuring QALY, but could serve as the final outcome measure for cost-utility analysis. Moreover, the utility weights can help patients make a decision regarding which clinical treatment would be a better choice. Hence, further studies using utility weights are needed. In the present study, we only estimated QALY losses due to CRC. If the decision makers would like to estimate the size of the BoD due to different neoplasms, the QALY should be evaluated according to each neoplasm. Moreover, QALY is a powerful measure for setting priorities for resource allocation among competing programs. Thus, QALY can be used to evaluate the outcome of CRC-related health programs.

In comparison with the results of QALY loss of other diseases in Korea^3^, we noticed that colorectal cancer have a significant burden of disease. In the previous study conducted by Ock *et al*.^3^, QALY losses in Korean adults due to 13 communicable diseases (except cancers) in 2010 were estimated. According to the Ock *et al*.^3^, QALY loss of hypertension (513,113) was largest, followed by arthritis (509,317) and stroke (431,049). Although the comparability is limited due to the different study period, colorectal cancer was ranked seventh in the 14 diseases. The burden of colorectal cancer estimated by QALY loss was larger than myocardial infarction, depression, and asthma, while it was smaller than diabetes mellitus and osteoporosis. These comparison results can be helpful in determining an appropriate priority for healthcare resource allocation.

In Korea, colorectal cancer mainly affected QALY loss due to mortality, considering the composition of QALY loss. About three-quarters of the total QALY loss (74.8%) was QALY loss due to mortality. Furthermore, QALY loss of colorectal cancer was larger in men than women and these differences were mainly attributed to high prevalence and mortality of colorectal cancer in 50 s and 60 s men groups. We expected that interventions to decrease the mortality and incidence of colorectal cancer in these vulnerable groups are preferentially required in order to reduce the burden of colorectal cancer.

Main limitation of this study was that we did not assess the degree of survey participants’ difficulty in conducting SG and verify the validity and reliability of participants’ responses from SG. The VAS was performed to familiarize the participants with the health state of CRC, before SG was applied and outlier VAS values for being dead (≥20) were excluded from the analyses to include only participants likely to understand the way of survey including valuation methods. Moreover, we excluded 54 responses because of their inconsistent responses. Despite all these efforts, we could not completely rule out the possibility that inappropriate responses were included in the analyses of utility weights.

Another limitation of our present study was that the calculation of total QALY loss due to CRC was restricted to individuals aged ≥30 years. Although this restriction was based on the availability of data of the prevalence of CRC from the NCCI, the estimated total loss due to CRC would be underestimated. However, considering the very low incidence of CRC in individuals aged <30 years, the degree of underestimation of total QALY loss due to CRC would be minimal. In addition, valuation on worse than HS was not considered because of reducing cognitive burden of study respondents. This also could underestimate the results. But, few people might value some HSs as a worse than death so its effect may be relatively small.

In conclusion, we determined the utility weights of HSs associated with CRC that reflect the societal preference of the Korean population and estimated the QALY loss due to CRC. The results from this study will be helpful for conducting cost-utility studies of healthcare interventions, such as cancer screening for CRC, and for measuring the BoD using QALY.

## Methods

**Figure 1:**
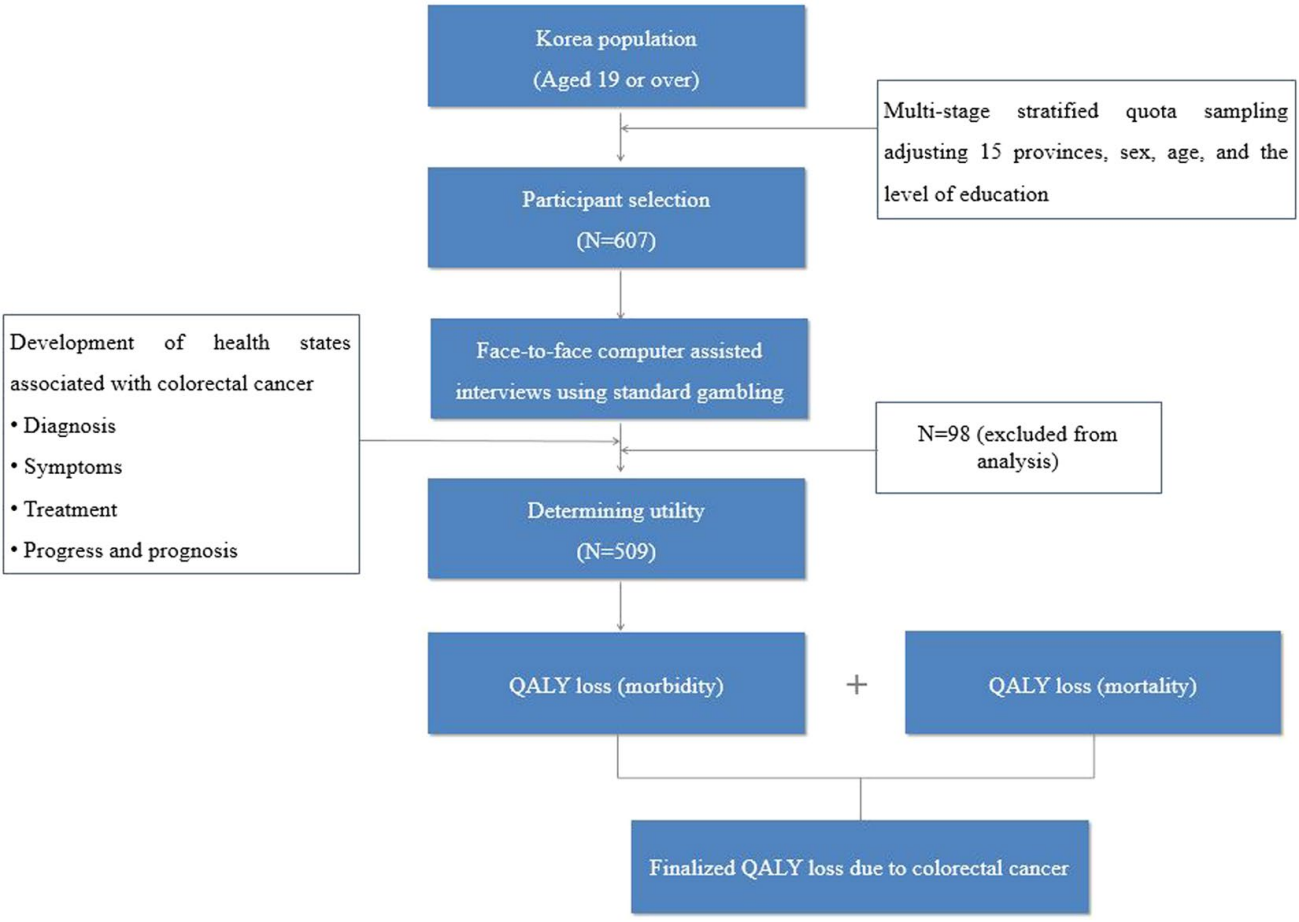
Study framework.

Figure 1 shows the framework of the study, wherein the utility weights for HSs associated with CRC were measured and were used to estimate the QALY loss.

### Developing the description of health states associated with CRC

The authors (Ock M, Jo MW, and Lee JY) developed a draft of the HSs associated with CRC based on expert interviews and literature reviews. After reviewing a draft from a surgeon (Lee JL; one of authors in this article), we finalized the description of HSs associated with CRC. We defined eight HS descriptions, from perfect health (HS 0) to metastatic CRC (HS 7); each HS description comprised four components: diagnosis, symptoms, treatment, and progress and prognosis (Table 4). The complete descriptions of the 8 HSs are provided in Supplement 1.

**Table 4:** Description of the health states associated with colorectal cancer.

HS	Diagnosis	Symptom	Treatment	Progress and prognosis
1	Adenomatous polyps	• Mostly asymptomatic	• Polyps can be removed during a colonoscopy under sedation	• Complications of colonoscopy: abdominal discomfort, small amount of bleeding, bowel perforation
		• Rarely bloody stool, mucous stool, and change in bowel habits can occur		• Rate of recurrence of adenomatous polyps: 30–50%,
				• Risk of progression to cancer: 1%
2	Colon cancer requiring colon resection	• Mostly asymptomatic	• Open or laparoscopy colectomy	• Complications of surgery: temporary change in bowel habits.
		• A change in bowel habits, anemia, mild dyspepsia, mucous stool, and bloody stool can occur		• Recurrence rate: 3–15%
				• 5-year survival rate: 85–97%.
3	Rectal cancer requiring resection of the rectum	• Mostly asymptomatic	• Upper rectum: open or laparoscopic resection of the rectum.	• Complications of surgery: temporary voiding dysfunction and sexual dysfunction
		• A change in the bowel habits and bloody stool can occur	• Lower rectum: open or laparoscopic resection of the rectum with sphincter-sparing operation	• Recurrence rate: 7–21%
				• 5-year survival rate: 79–94%.
4	Colon cancer requiring colon resection and systemic chemotherapy	• A change in bowel habits, anemia, blood in stool, mucous stool, and abdominal discomfort or pain can occur.	• Open surgery to remove the cancer	• Complications of surgery: temporary change in bowel habits
		• Feeling of a lump in the abdomen.	• Systemic chemotherapy to reduce recurrence	• Problems in sleeping, and the feeling of fear or anxiety
				• Recurrence rate: 17–56%
				• 5-year survival rate: 44–83%.
5	Rectal cancer requiring resection of the rectum and chemoradiation therapy	• A change in bowel habits may occur.	• Neoadjuvant chemoradiation for 6 weeks,	• Complications of surgery: temporary voiding dysfunction and sexual dysfunction
		• Anal pain, blood in the stool, and abdominal discomfort due to dyspepsia may occur.	• Surgical resection and adjuvant systemic chemotherapy for 6 months	• Problems in sleeping, and the feeling of fear or anxiety about illness
				• Recurrence rate: 20–55%
				• 5-year survival rate: 45–80%
6	Rectal cancer requiring resection of the rectum, stoma formation, and chemoradiation therapy	• A change in bowel habits may occur.	• Chemoradiation for 6 weeks	• Complications of surgery: temporary voiding dysfunction and sexual dysfunction
		• Anal pain, blood in the stool, and abdominal discomfort due to dyspepsia and anal mass may occur.	• Surgical resection with stoma formation	• Patients need to defecate through the stoma
			• Adjuvant systemic chemotherapy for 6 months	• Problems in sleeping, and the feeling of fear or anxiety about illness
				• Recurrence rate: 20–55%
				• 5-year survival rate: 45–94%
7	Metastatic colon cancer	• Blood in stool, anemia, abdominal pain, bowel obstruction, jaundice, and/ or ascites	• Surgery and consequent systemic chemotherapy	• Complications of surgery: temporary voiding dysfunction and sexual dysfunction
		• Rarely mobility impairment, pain, and paralysis may occur due to spinal metastasis	• Radiation therapy to treat bony metastasis lesions in the inoperable area	• Patients need to defecate through the stoma, if surgery is performed
			• Systemic chemotherapy for the remaining metastatic lesions	• Problems in sleeping, and the feeling of fear or anxiety about illness
				• 5-year survival rate: 6–30%

HS: health state.

### Survey participants

We recruited a total of 607 representative individuals using a multistage stratified quota sampling method. The quota was assigned to each of 15 provinces based on their population proportion, along with gender, age group, and education level. These data were derived from the resident registration information, as of June 2013, which was published by the Ministry of Administration and Security in Korea.

### Face-to-face computer-assisted interviews by trained interviewers

The survey was performed by trained interviewers via face-to-face computer-assisted interviews. The trained interviewers had learned about the CRC-related HSs and were trained on how to perform the SG technique. The interviewers practiced in pairs before performing the field survey. The total training time for each interviewer was approximately 2.5 hours. The overall interview process was as follows: interviewers asked the participants about their general characteristics such as gender, age, and education level, and then helped the participants evaluate each HS of CRC using the VAS and SG approach. To ensure that participants understood the HSs, the interviewers used visual aids.

### Process for determining utility weights for CRC-related health states using SG

**Figure 2:**
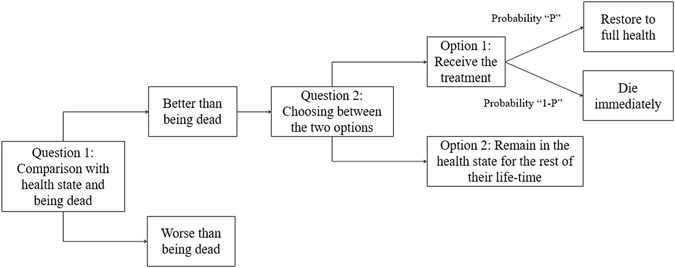
Flow chart of standard gamble.

Before SG was applied, the VAS was administered to participants, so that they could familiarize themselves with the HSs evaluated. The participants were administered the VAS a total of five times, for which four HSs were randomly selected among the seven HSs and the “being dead” HS. Thereafter, SG was applied for the participants. In SG, the participants need to decide between two HSs, one of which is a selected hypothetical HSi (i is a certain HS) and the other is death (Question 1 in Fig. 2). Thereafter, they had to choose between the following 2 options (Question 2 in Fig. 2): first, they could remain in the HSi for the rest of their life-time (Option 2 in Fig. 2) or they could choose to receive treatment (Option 1 in Fig. 2) that may either restore them to full health (probability, “P”) or immediately kill them (probability, “1 - P”). The participants continued to make choices until the preference for the two choices became equal. The minimum probability interval was 5%. The chances of achieving the best outcome started at 50%, and increased or decreased by 5% according to the individual’s response.

### Analysis of utility weight

After applying the SG approach to all participants, we collected all the answers. Thereafter, the utility weight for each HS was calculated as the possibility of restoring full health (if the HS was regarded as a better option than being dead). However, in cases where the HS was regarded as worse than being dead, the utility weight was censored at zero. We excluded 44 participants with outlier VAS values for being dead (≥20) from the analyses. In addition, 54 participants who had more than four inconsistent responses were also excluded in the SG approach. We assumed that a response was inconsistent when the HS 1′s utility weight was lower than other HSs’ utility weight or HS 7′s utility weight was higher than other HSs’ utility weight. The mean values for the utility weights according to the socio-demographic factors and clinical information were compared using Student’s t-test and analysis of variance (ANOVA). All statistical analyses were conducted using SPSS software (v21.0). A P-value of <0.05 was considered statistically significant.

### Calculation of QALY loss

We estimated the total QALY loss due to CRC in Korean individuals aged ≥30 years in 2013, by using the calculated utility weights from this study and the methods from a previous study^3^. The total QALY loss due to a certain condition is the sum of the QALY losses due to morbidity and mortality. The details of the method for the calculation of QALY loss can be found elsewhere^3^. In order to calculate the QALY loss due to morbidity, we needed two data sets: the prevalence rate and the utility weights for CRC.

With regard to the prevalence rate, we used data from the National Cancer Control Institute (NCCI). NCCI provides cancer statistics in Korea, including the incidence and mortality rates for all cancers by severity level, gender, and age group. The severity level was classified according to the SEER stage, as follows: localized stage, regional stage, distant stage, and unknown stage. Based on the data for incidence and mortality rates for colon cancer, we estimated the prevalence rates for colon cancer by SEER stage, gender, and age group^27^.

With regard to the utility weights for CRC, the average utility weight for HS 2 and 3 in this study was considered as the utility weight for the localized stage. Moreover, the average utility weight for HS 4, 5, and 6 was considered as the utility weight for the regional stage. In addition, the utility weight for HS 7 was mapped as the utility weight for the distant stage, whereas the mean utility weight for HS 6 and 7 was considered as the utility weight for the unknown stage. Furthermore, we assumed the utility weight for healthy controls as 1 (full health).

We conducted sensitivity analyses considering unknown stage. We assumed that CRC patients of unknown stage might be mainly mixed group of regional and distant stage. In order to estimate its effect on QALY loss, sensitivity analyses were done by assuming unknown stage as a separate stage or other stages. We also performed sensitivity analyses by applying a 5%, 10%, or 20% increase in the prevalence of CRC.

The age group interval was five years, and the final age group was ≥80 years. In order to calculate the QALY loss due to mortality, we obtained mortality and life expectancy data in 2013 from the Korean Statistical Information Service from Statistic Korea.

### Ethics statement

This study was approved by the institutional review board of Asan Medical Center (S2014-1396-000), and informed consent was obtained from all participants. This study was performed in accordance with relevant guidelines and regulations of the institute review board of Asan Medical Center.

## Electronic supplementary material

Supplementary information
