# Supplementary material for: Estimating utility weights and quality-adjusted life year loss for colorectal cancer-related health states in Korea
Source: Sci Rep. 2017 Jul 17;7:5571. doi: 10.1038/s41598-017-06004-6 (PMC5514107; doi:10.1038/s41598-017-06004-6)
Supplement: Supplementary file 1 — Supplementary information [file 41598_2017_6004_MOESM1_ESM.doc]

**Estimating utility weights and quality-adjusted life year loss for colorectal cancer-related health states in Korea**

Jin Yong Lee^1,2,†^, Minsu Ock^3,†^, Min-Woo Jo^3^,*, Woo-Seung Son^3^, Hyeon-Jeong Lee^3^, Seon-Ha Kim^4^, Hyun Joo Kim^5^ & Jong Lyul Lee^6^

Supplemental Table 1. QALY loss due to morbidity on sensitivity analysis considering unknown stage

|  | QALY loss due to morbidity | | | | | |
| --- | --- | --- | --- | --- | --- | --- |
|  | Men | | | Women | | |
| age group | Unknown stage was considered as a separate stage | Unknown stage was assumed as a localized stage | Unknown stage was assumed as a distant stage | Unknown stage was considered as a separate stage | Unknown stage was assumed as a localized stage | Unknown stage was assumed as a distant stage |
| 30-34 | 261 | 253 | 264 | 200 | 195 | 203 |
| 35-39 | 554 | 535 | 562 | 391 | 381 | 396 |
| 40-44 | 1,000 | 972 | 1,012 | 739 | 723 | 746 |
| 45-49 | 1,695 | 1,649 | 1,715 | 1,184 | 1,154 | 1,197 |
| 50-54 | 3,194 | 3,120 | 3,228 | 1,985 | 1,945 | 2,003 |
| 55-59 | 3,758 | 3,674 | 3,795 | 1,951 | 1,909 | 1,970 |
| 60-64 | 4,223 | 4,131 | 4,264 | 2,040 | 1,997 | 2,059 |
| 65-69 | 4,130 | 4,039 | 4,170 | 2,292 | 2,247 | 2,311 |
| 70-74 | 3,912 | 3,826 | 3,950 | 2,513 | 2,455 | 2,539 |
| 75-79 | 2,354 | 2,285 | 2,384 | 2,044 | 1,976 | 2,074 |
| 80+ | 1,483 | 1,395 | 1,522 | 1,909 | 1,762 | 1,973 |
| Total | 26,564 | 25,878 | 26,867 | 17,248 | 16,745 | 17,470 |

Supplemental Table 2. QALY loss due to morbidity on sensitivity analysis considering an increase in the prevalence of CRC

|  | QALY loss due to morbidity | | | | | | | |
| --- | --- | --- | --- | --- | --- | --- | --- | --- |
|  | Men | | | | Women | | | |
| age group | Original value | 5% increase | 10% increase | 20% increase | Original value | 5% increase | 10% increase | 20% increase |
| 30-34 | 261 | 274 | 287 | 313 | 200 | 210 | 220 | 241 |
| 35-39 | 554 | 582 | 609 | 665 | 391 | 411 | 430 | 469 |
| 40-44 | 1,000 | 1050 | 1100 | 1200 | 739 | 776 | 813 | 887 |
| 45-49 | 1,695 | 1780 | 1864 | 2034 | 1,184 | 1243 | 1302 | 1420 |
| 50-54 | 3,194 | 3354 | 3514 | 3833 | 1,985 | 2084 | 2184 | 2382 |
| 55-59 | 3,758 | 3946 | 4134 | 4510 | 1,951 | 2049 | 2146 | 2341 |
| 60-64 | 4,223 | 4435 | 4646 | 5068 | 2,040 | 2142 | 2244 | 2448 |
| 65-69 | 4,130 | 4336 | 4543 | 4956 | 2,292 | 2406 | 2521 | 2750 |
| 70-74 | 3,912 | 4107 | 4303 | 4694 | 2,513 | 2639 | 2764 | 3016 |
| 75-79 | 2,354 | 2472 | 2589 | 2825 | 2,044 | 2146 | 2248 | 2452 |
| 80+ | 1,483 | 1557 | 1631 | 1779 | 1,909 | 2004 | 2099 | 2290 |
| Total | 26,564 | 27,892 | 29,220 | 31,876 | 17,248 | 18,110 | 18,973 | 20,697 |

**Supplemental Table 3**. Number of inconsistent response in standard gamble

| Inconsistent responses | | N | | % |
| --- | --- | --- | --- | --- |
| 0 | 419 | | 69.0 | |
| 1 | 59 | | 9.7 | |
| 2 | 37 | | 6.1 | |
| 3 | 31 | | 5.1 | |
| 4 | 46 | | 7.6 | |
| 5 | 8 | | 1.3 | |
| 6 | 3 | | 0.5 | |
| 7 | 4 | | 0.7 | |

**Supplement 1. Full description of the health states associated with colorectal cancer**

**1) Adenomatous polyps**

• Diagnosis: You have recently been diagnosed with adenomatous polyps by colonoscopy and biopsy.

• Symptoms: In most cases, adenomatous polyps are asymptomatic, but a large polyp can cause a change in bowel habits, bloody stool and mucous stool.

• Treatment: Polyps can be removed during a colonoscopy. Mostly, you will not feel any pain or awareness since this procedure is performed under appropriate sedation.

• Progress and prognosis of the disease: In rare cases, there can be lower abdominal discomfort and small amount of bleeding after the removal of polyps, which will be improved within 3 to 4 hours. About 0.3%~1.0% of patients can have complications of bowel perforation. If this happens to you, a surgery can be required. Recurrence rate is 30~50%, and the risk of progression to cancer is 1%, which is higher than the general population.

**2) Colon cancer requiring colon resection**

• Diagnosis: You have recently been diagnosed with colon cancer requiring colon resection.

• Symptoms: In most cases, this stage of colon cancer is asymptomatic but there could be symptoms like a change in bowel habits, anemia, mild dyspepsia, mucous stool and bloody stool.

• Treatment: You will have surgery to remove the part of your colon that contains the cancer by open or laparoscopic approach.

• Progress and prognosis of the disease: Bowel habits can be changed temporarily after the surgery, but it will return to normal within 1 year. Recurrence rate after surgery is 3~15%. You will be observed by regular follow-up visits. In these appointments, you will get blood tests, chest X-rays, colonoscopy, and CT imaging every 3 to 6 months. Your 5-year survival rate is about 85~97%.

**3) Rectal cancer requiring resection of the rectum**

• Diagnosis: You have recently been diagnosed with rectal cancer requiring resection of the rectum.

• Symptoms: In most cases, this stage of rectal cancer is asymptomatic, but it can cause a change in the bowel habits and bloody stool.

• Treatment: Cancer involving upper rectum needs resection of the rectum only. However, that involving lower rectum needs sphincter-sparing operation in addition to the rectal resection. Surgery can be performed by open procedure or by laparoscopic approach.

• Progress and prognosis of the disease: Voiding dysfunction and sexual dysfunction can occur temporarily after the surgery, but you will recover soon. Recurrence rate after surgery is 7 to 21%. You will be observed by regular follow-up visits. In these appointments, you will get blood tests, chest X-rays, colonoscopy, CT, MRI and PET imaging every 6 to 12 months. Your 5-year survival rate is about 79 to 94 %.

**4) Colon cancer requiring colon resection and systemic chemotherapy.**

• Diagnosis: You have recently been diagnosed with colon cancer requiring colon resection and systemic chemotherapy.

• Symptoms: This stage of colon cancer can cause a change in your bowel habits including diarrhea or constipation, or a change in the consistency of your stool. Also, it may cause anemia, blood in your stool and mucous stool. Abdominal discomfort or pain can be present. Sometimes, you can feel a lump in your abdomen.

• Treatment: You will have open surgery to remove the cancer. Then, you will receive systemic chemotherapy in a purpose of reducing the recurrence. You have to visit the hospital 2 to 3 times every 2 to 4 weeks until 6 months to get chemotherapeutic infusion. You may feel weakness or fatigue for a few days after the infusion which may interrupt your daily living. Your skin may turn dark or can be peeled off because of drug’s side effects, but you can recover after finishing the treatment.

• Progress and prognosis of the disease: A change in bowel habits can occur temporarily after the surgery, but it will be normalized within 1 year. The feelings of fear or anxiety may present due to worries about the illness and death, and sometimes you can have problems in sleeping. Recurrence rate after surgery is 17 to 56%. You will be observed by regular follow-up visits. In these appointments, you will get blood tests, chest X-rays, colonoscopy, CT, and PET imaging every 3 to 6 months. Your 5-year survival rate is about 44 to 83%.

**5) Rectal cancer requiring resection of the rectum and chemoradiation therapy.**

• Diagnosis: You have recently been diagnosed with rectal cancer requiring resection of the rectum and chemoradiation therapy.

• Symptoms: This stage of rectal cancer can cause a change in your bowel habits including diarrhea or constipation, or a change in the consistency of the stool. Anal pain, blood in the stool and abdominal discomfort due to dyspepsia may occur as well.

• Treatment: Before surgery, you will receive chemoradiation therapy for 5 to 6 weeks. During this period, you need to visit the outpatient clinic for 5 times a week. You may feel discomfort in your pelvis and pain around your anus after the treatment. Also, the treatment can cause symptoms of urinary frequency and painful voiding, but you will recover soon after finishing the treatment. By the time you are done with radiation therapy, you will have a surgery, and then you will receive systemic chemotherapy. You have to visit the hospital 2 to 3 times every 2 to 4 weeks until 6 months to get infusion of chemotherapeutic agents. You may feel weakness or fatigue for a few days after the infusion that may interrupt your daily living. Your skin may turn dark or can be peeled off because of drug’s side effects, but you can recover after finishing the treatment.

• Progress and prognosis of the disease: Voiding dysfunction and sexual dysfunction can occur temporarily after the surgery, but you will recover soon. Recurrence rate after surgery is 20 to 55%. The feelings of fear or anxiety may present due to worries about the illness and death, and sometimes you can have problems in sleeping. You will be observed by regular follow-up visits. In these appointments you will get blood tests, chest X-rays, colonoscopy, CT, MRI and PET imaging every 6 to 12 months. Your 5-year survival rate is about 45 to 80 %.

**6) Rectal cancer requiring resection of the rectum, stoma creation and cheomoradiation therapy.**

• Diagnosis: You have recently been diagnosed with rectal cancer requiring resection of the rectum, stoma creation and cheomoradiation therapy.

• Symptoms: This stage of rectal cancer can cause a change in your bowel habits including diarrhea or constipation, or a change in the consistency of the stool. Anal pain, blood in the stool and abdominal discomfort due to dyspepsia may occur as well. You may find a mass in your anus.

• Treatment: Before surgery, you will receive chemoradiation therapy for 5 to 6 weeks. During this period, you need to visit the outpatient clinic for 5 times a week. You may feel discomfort in your pelvis and pain around your anus after the treatment. Also, the treatment can cause symptoms of urinary frequency and painful voiding, but you will recover soon after finishing the treatment. You will have open surgery, which will remove your rectum and create a stoma in your abdomen. Then, you will receive systemic chemotherapy. You have to visit the hospital 2 to 3 times every 2 to 4 weeks until 6 months to get infusion of chemotherapeutic agents. You may feel weakness or fatigue for a few days after the infusion that may interrupt your daily living. Your skin may turn dark or can be peeled off because of drug’s side effects, but you can recover after finishing the treatment.

• Progress and prognosis of the disease: You will defecate through a stoma in your abdomen, which is an artificial anus. You always have to carry a stool pouch over the stoma. You need to take care of this pouch all the time and this will be disturbing to your daily living. Sometimes it may smell bad. You have to change the pouch regularly, one or two times per week. The feelings of fear or anxiety may present due to worries about the illness and death, and sometimes you can have problems in sleeping. Voiding dysfunction and sexual dysfunction can occur temporarily after the surgery, but you will recover soon. Recurrence rate after surgery is 20 to 55%. You will be observed by regular follow-up visits. In these appointments you will get blood tests, chest X-rays, colonoscopy, CT, MRI and PET imaging every 6 to 12 months. Your 5-year survival rate is about 45 to 94%.

**7) Metastatic colon cancer**

• Diagnosis: You have recently been diagnosed with colon cancer metastasized to lungs and liver.

• Symptoms: This stage of colon cancer can cause blood in stool, anemia, abdominal pain, bowel obstruction, jaundice and/or ascites. Rarely, vertebral bones can be damaged due to spinal metastasis. In that case, mobility impairment, back pain and sometimes paralysis may occur.

• Treatment: If surgical resection is possible, you will have surgery first and then receive consequent systemic chemotherapy. Radiation therapy can be used to treat bone metastasis or lesions in the inoperable area of your body. In the case of peritoneal metastasis or severe obstructive bowel symptoms you may need to remove a part of your bowel and create a stoma. If there are any metastatic lesions remaining, systemic chemotherapy is recommended and you could try targeted therapy. You may feel weakness or fatigue for a few days after the infusion that may interrupts your daily living. Your skin may turn dark or can be peeled off because of drug’s side effects, but you can recover after finishing the treatment.

• Progress and prognosis of the disease: If you have surgery, a change in bowel habits can occur after the surgery. If a surgery creates a stoma, you will defecate through a stoma in your abdomen, which is an artificial anus. You always have to carry a stool pouch over the stoma. You need to take care of this pouch all the time and this will be disturbing to your daily living. Sometimes it may smell bad. You have to change the pouch regularly, one or two times per week. The feelings of fear or anxiety may present due to worries about the illness and death, and sometimes you can have problems in sleeping. If you have surgery, voiding dysfunction and sexual dysfunction can occur temporarily. Your 5-year survival rate is about 6 to 30%.
